# Mitral Valve Prolapse in Pregnancy

**DOI:** 10.5935/1678-9741.20160034

**Published:** 2016

**Authors:** Shi-Min Yuan, Song-Li Yan

**Affiliations:** 1 The First Hospital of Putian, Teaching Hospital, Fujian Medical University, Fujian Province, China.

**Keywords:** Mitral Valve Insufficiency, Mitral Valve Prolapse, Pregnancy

## Abstract

Mitral valve prolapse is a benign condition. Mitral regurgitation is only complicated in patients with severe mitral valve prolapse. Women with mitral valve prolapse in the absence of other cardiovascular disorders tolerate pregnancy well and do not develop remarkable cardiac complications. Nevertheless, serious complications of mitral valve prolapse, including arrhythmia, infective endocarditis and cerebral ischemic events, can be present in pregnancy. Debates remain with regard to the use of prophylactic antibiotics and β-blockers in the pregnant women with mitral valve prolapse. The prognosis of the pregnant patients might be closely related to the pathological and (or) functional changes of the mitral valve. Non-myxomatous mitral valve prolapse poses no or little obstetric risks in terms of pregnancy, labor and neonatal complications; whereas myxomatous mitral valve prolapse is a major etiology of valvular heart disease in women of childbearing age. In the pregnant patients with mitral valve prolapse progressing into major complications, surgical interventions are considered. Medicinal treatment of such patients with β-blockers should be a concern for the fetal safety.

**Table:** 

Abbreviations, acronyms & symbols	
MR	= Mitral regurgitation
MVP	= Mitral valve prolapse

## INTRODUCTION

Mitral valve prolapse (MVP) is defined as a prolapse of one or both mitral valve leaflets at least 2 mm beyond the long axis annular plane with or without mitral thickening^[1]^. MVP is characterized by elongated chordae tendineae and redundant valve leaflets, which prolapse into the left atrial cavity as the ventricle contacts. The prolapse may or may not result in mitral regurgitation (MR)^[2]^. It is uncommon that the patients are complicated with severe sequelae, the most frequent of which are severe MR that usually warrants a surgical correction^[3]^. MVP may be due to a primary connective tissue disease involving the mitral valve leaflets, the subvalvular apparatus, or the mitral annulus, or secondary to mitral valve apparatus abnomalities. Primary or idiopathic MVP is usually associated with myxomatous, redundant valve leaflets and progressive annular dilation. In contrast, patients with secondary MVP have thin leaflets, which prolapse slightly into the left atrium during systole as a result of mismatch of the anatomical relationship between the mitral valve apparatus and left ventricle. Hence, secondary MVP may be seen in secundum atrial septal defects, infective endocarditis, rheumatic mitral stenosis and calcified mitral annulus^[4]^. The reason of the mucopolysaccharide accumulation predilection in the mitral valve remains uncertain. MVP was also regarded as an autosomal dominant cardiac abnormality with age and sex dependent expression^[5]^. Nevertheless, it is believed that MVP is a result of progressive myxomatous valve changes and it may bring about chordal rupture in some patients^[6]^. The progression of MVP into severe MR usually occur after the age of 50 years^[3]^; whereas younger patients may have good exercise tolerance, and would not show any circulatory deterioration^[4]^.

Cardiac problems in pregnancy have been a contemporary important topic of concern^[7]^. The incidence of cardiac disease in the pregnant women was estimated to be 0.5%, of which mitral valve disease was more common than aortic (94.5% vs. 5.5%), and MVP was more common (39.2%) than MR (19.9%), mitral stenosis (16.9%), or combined valvular disorders (24%^[8]^. As reported by Nanna & Stergiopoulos^[9]^, MVP (myxomatous changes) was a major etiology of valvular heart disease in women of childbearing age. Gelson et al.^[10]^ reported that MVP is the most common cardiac disorder in the pregnant population, accounting for 12-17% of women of childbearing age. Women with MVP in the absence of other cardiovascular disorders may tolerate pregnancy well and do not develop remarkable cardiac complications. Although rare, more serious complications of MVP, such as arrhythmia, infective endocarditis and cerebral ischemic events, have been reported during pregnancy^[4]^. Debates still remain in the management of MVP in the pregnant patients. This article aimed to delve deeper into this topic.

## CLINICAL FEATURES

The routine use of echocardiography has greatly facilitated the identification of MVP in young adults. Symptoms are variable, but the most frequent complaints are dizziness, palpitation and faintness^[11]^. Disappearance of auscultatory findings, midsystolic click and late systolic murmur of MR may pose a special problem in diagnosis of MVP during pregnancy^[12]^. Perhaps these changes leave many cases of MVP undetected leading to false reduction in the incidence of MVP during pregnancy^[13]^. Patients with MVP may have MR and benign ventricular ectopics. Anatomic findings of MVP include 1) mitral annular dilation, 2) redundant and thickened leaflets, 3) myxomatous transformation of valve substance due to collagen dissolution or disruption and severe acid mucopolysaccharide accumulation, and 4) absence of inflammatory changes^[4]^. However, these patients may not show any deterioration throughout pregnancy^[4]^. MVP with diffusely thickened, redundant leaflets (myxomatous change) are more prone to deformity, degeneration and infection compared to those with only MVP without thickened leaflets (non-myxomatous changes)^[14]^. Recognizing and diagnosing MVP will explain episodes of dyspnea, palpitation and chest pain during pregnancy. Most obstetric patients with MVP are in the low risk group, i.e., the non-myxomatous form, and should be expected to have excellent obstetric outcomes.

**Fig. 1:**
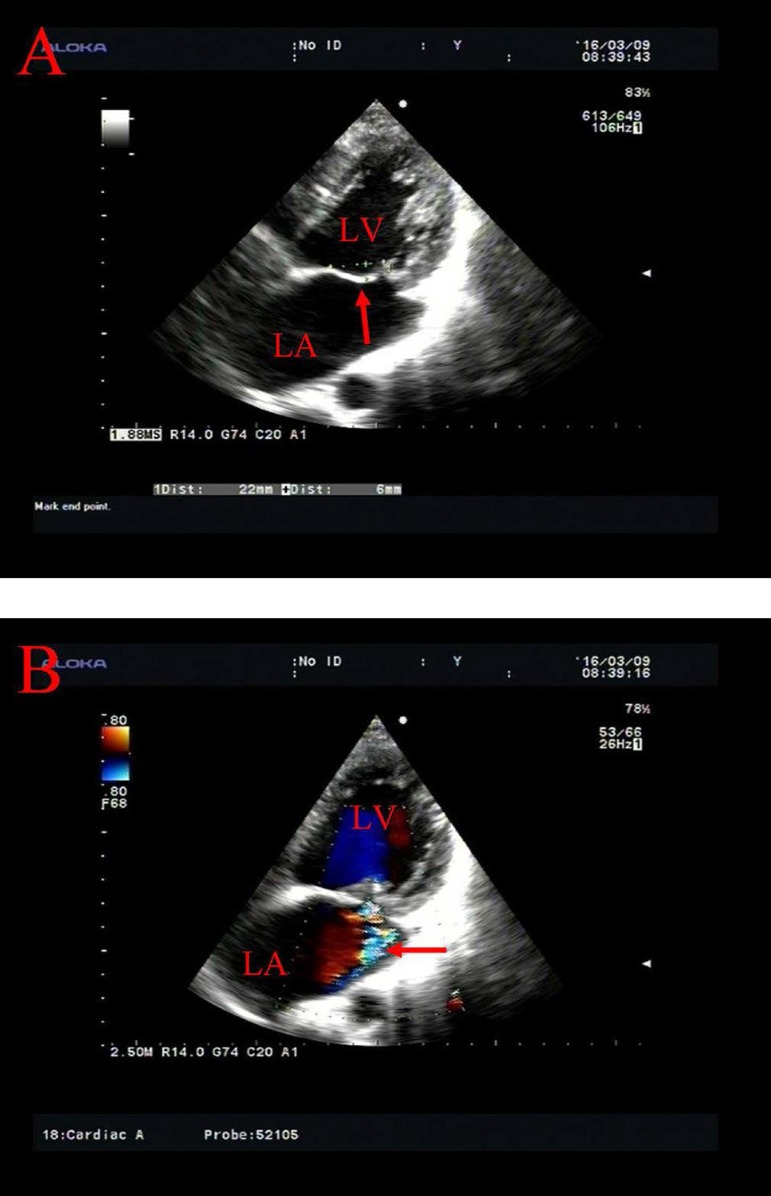
Transthoracic echocardiography of mitral valve prolapse. (A) Prolapse of the anterior mitral leaflet with movement of the coaptation behind the annular plane (arrow); (B) Mitral insufficiency with a regurgitant flow along the posterior wall of the left atrium (arrow). LA=left atrium; LV=left ventricle

Echocardiography is of special significance for the diagnosis of MVP. The parasternal long-axis view on transthoracic echocardiography may illustrate whether the mitral leaflets are prolapsed into the left atrial cavity by surpassing the connecting line between the annulus of the anterior and posterior leaflets^[15]^. Besides, obvious balloon-like changes, leaflet thickening and elongation, annular dilation, left atrial and left ventricular enlargement, and diminution, elongation, or rupture of chordae tendineae could be seen on two-dimensional echocardiography. On M-mode echocardiography, posterior displacement of mitral valve coaptation line (CD segment) is > 2 mm during late systolic phase, and > 3 mm during pansystolic phase. Meanwhile, one or two leaflets may show hammock-like changes^[15]^. In patients with prolapse of the anterior mitral leaflet, the mitral regurgitant flow could be seen along the posterior wall of the left atrium (Figure 1); while in those with prolapse of the posterior mitral leaflet, the mitral regurgitant flow could be seen along the anterior wall of the left atrium^[16]^.

## MATERNAL PROGNOSIS

The severity of prolapse depends on factors that reduce ventricular volume, which is in an inverse proportion to the extent of MVP. The smaller the ventricular volume, the greater the prolapse^[2]^. Although the course and the prognosis of MVP are often benign, it may be complicated infrequently by progressive MR, ruptured chordae tendineae, infective endocarditis, transient ischemic attacks, malignant arrhythmias, or sudden death^[17]^. There have also been a few case reports regarding more serious complications associated with MVP during pregnancy, such as cerebrovascular accident^[18]^, thromboembolic ischemia^[19]^ and infective endocarditis^[20]^. Sugrue et al.^[21]^ reported a case of postpartum group B streptococcal endocarditis developed following uncomplicated spontaneous vaginal delivery. Echocardiography showed moderate to severe MR, and vegetation on the atrial surfaces of both leaflets^[20]^. More definitive recommendations regarding prophylaxis for MVP and uncomplicated vaginal delivery await further study^[4]^. Sugrue et al.^[21]^ proposed that routine peripartum antibiotic prophylaxis was not indicated for the prevention of infective endocarditis and prophylaxis should only be given to a complicated delivery and for patients with MR. However, infective endocarditis and asymptomatic puerperal bacteremia after normal vaginal delivery are uncommon. There is evidence that antibiotic prophylaxis may increase the risk of refractory endocarditis, and recommended prophylactic regimens carry a considerable risk of drug toxicity.

## FETAL OUTCOME

Several studies have examined the possible association between MVP and pregnancy outcomes. Maternal valvular heart diseases have been associated with lower Apgar scores, increased preterm delivery rate, intrauterine growth restriction and reduced birth weight in the newborn^[22]^. The risk to both the mother and the fetus might get exponentially increased with the complexity of the valvular disorders, such as hemodynamic status, heart function class, cyanotic manifestations and pulmonary artery hypertension^[23]^. Chia et al.^[4]^ investigated pregnancy outcomes in 28 patients with echocardiographic evidence of non-myxomatous MVP, all the patients remained well during pregnancy. Tang et al.^[24]^ examined 37 pregnancies in women with MVP with no cardiac complications or maternal mortality associated with either vaginal or cesarean delivery. Chen et al.^[25]^ conducted a large-scale study to evaluate pregnancy outcomes of 3,104 women with MVP, and found higher rates of preterm birth and cesarean section in a multivariate model. No association between mitral valve disorder and recurrent abortions was noted. A possible explanation might be an increase in platelet activity in the presence of MR^[26]^.

Patients with non-myxomatous MVP showed much better prognosis than those with myxomatous MVP as evidenced by echocardiography^[4]^. Non-myxomatous MVP presents little or no obstetric risk in terms of pregnancy, labor and neonatal complications, with a cesarean section rate of 14.3% and excellent neonatal outcomes^[4]^. In terms of adverse pregnancy outcomes, Chen et al.^[25]^ found that mothers with MVP were significantly more likely to have neonates with low body weight, preterm birth and cesarean delivery, and found that the distributions of outcomes including intrapartum complications, low Apgar scores, and congenital malformation did not differ significantly between women with and those without MVP. Women with an MVP were significantly at a 1.27- and 1.34-fold increased risk of having preterm births and cesarean deliveries, respectively. The link between MVP and preterm delivery was postulated as a result of discordant muscle traction, or lower muscle tone^[25]^. The discordant muscle traction, especially during pregnancy, might synchronously affect both cardiac papillary and uterine cervical muscles^[25]^. The characterized collagen dissolution of the mitral valve with secondary myxomatous transformation supported this hypothesis^[13]^.

## TREATMENT

The pregnancy-related cardiovascular changes often aggravate the clinical manifestations in the pregnant patients with valvular diseases. Female patients with severe valvular disorders should avoid pregnancy, due to the concern of high risks of maternal and fetal morbidity and mortality rates. In the patients with mild valvular lesions, pregnancy can be preceded under close monitoring by a sophisticated multidisciplinary team including the obstetrician, cardiologist and obstetric anesthesiologist^[27]^. Due to a drop in systemic vascular resistance and a decrease of left ventricular afterload, MR is often well-tolerated during pregnancy^[28]^. The pregnant patients with mild MR, normal left ventricular function without clinical symptoms are at low risks of pregnancy-associated complications. Asymptomatic women with non-myxomatous MVP with trivial or no MR probably do not require routine antibiotic prophylaxis and have excellent prognosis^[4]^; while symptomatic heart failure can be treated with nitrates, hydralazine, diuretics and digoxin^[10]^. Patients with arrhythmias, chest pain and palpitations often require β-blockers, such as propranolol^[13]^. If the pregnant patients progress into left ventricular dysfunction, they may develop pulmonary congestion, for which a restricted activity, a low-sodium intake and medical treatment with diuretics, β-blockers and vasodilators, are necessary. Though propranolol is the drug of choice in this condition, unfortunately, it might be of fetal hazards by readily crossing the placenta, and its safety during first trimester has been suspected. It causes an increase in uterine tone, which can potentially lead to a small infarcted placenta and a low-birth-weight infant through the β-blocking action on the uterus. Propranolol works directly as a β-blocker and also has a quinidine-like action. The use of propranolol at or close to the time of delivery can cause adverse neonatal effects, such as apnea, respiratory distress, bradycardia and hypoglycemia^[29]^. Growth retardation was noted in babies receiving atenolol in the first trimester, β-blockers should therefore be avoided in the first trimester and cardioselective β_1_-blockers, such as atenolol, are the preferred agents for the second and third trimesters, as they may interfere less with β_2_-mediated peripheral vasodilatation and uterine relaxation^[2]^_._ Moreover, vasodilators should be cautiously used in the pregnant patients, concerning the potential uteroplacental hypoperfusion^[9]^. If vasodilators are required for symptomatic relief, hydralazine and nitrates can be used, instead^[30]^. Angiotension-converting enzyme inhibitors are contraindicated in pregnancy and should be avoided^[30]^.

**Fig. 2:**
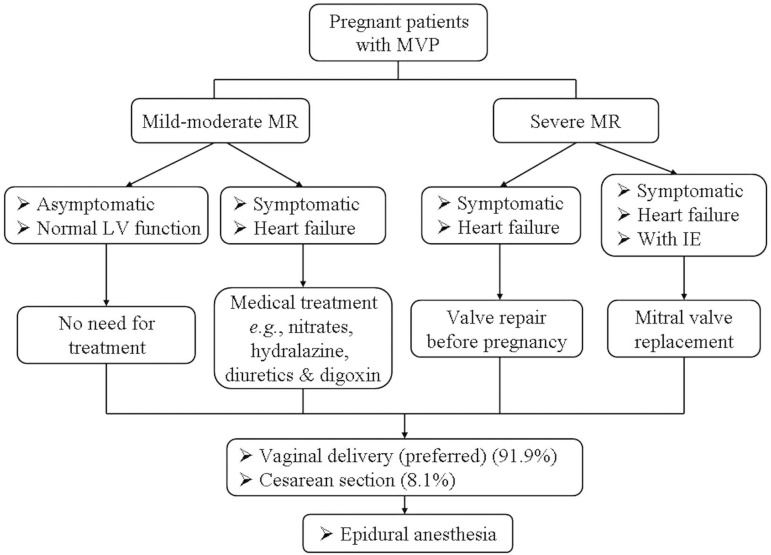
The management strategies of mitral regurgitation due to mitral valve prolapse in pregnant patients^[9,10,30]^. IE=infective endocarditis; LV=left ventricle; MR=mitral valve regurgitation; MVP=mitral valve prolapse

The female patients with severe MR and significant symptoms, or left ventricular functional impairment, warrant a valvular operation before a planned pregnancy, favorably with a valve repair procedure. Due to the fact that MVP is often a result of elongated or ruptured chordae tendineae, correction of MVP can be resorted to chordae replacement with mold pre built bovine pericardial chords (Braile-Gregori prosthesis) in addition to the rigid prosthetic semicircular ring (Gregori-Braile ring) for correction of mitral annulus dilation, in the replace of the widely accepted valve replacement^[31]^. The management strategies of MR due to MVP in pregnant patients were summarized in Figure 2. Surgical treatment of severe MR should be avoided concerning the materno-fetal risks during pregnancy except for the life-threatening infective endocarditis. In such cases, vaginal delivery is the preferred mode of delivery^[9]^.

## ANESTHESIA

Regional anesthesia is usually avoided because of the sympathetic denervation resulting in increased venous capacity and decreased peripheral resistance. This leads to a reduction of ventricular volume, which may increase the degree of prolapse. However, the safe use of epidural anesthesia in a patient with MVP has been reported^[32]^. It is quite possible that the majorities remain undiagnosed during pregnancy and undergo regional anesthesia without any untoward hemodynamic side effects^[2]^. Current American Heart Association guidelines stipulate that the presence of a murmur or echocardiographically demonstrated MR in patients with MVP constitutes a moderate risk of endocarditis with invasive procedures^[2]^.

## CONCLUSION

Women with MVP in the absence of other cardiovascular problems may tolerate pregnancy well. In the pregnant patients with MVP progressing into major complications, surgical interventions should be considered. Medicinal treatment in such patients with β-blockers should be a concern for the fetal safety. In the patients with severe MR and left ventricular fuctional impairment or New York Heart Association function class III or IV, delivery should be with the aid of carefully titrated epidural anesthesia and invasive hemodynamic monitoring.

**Table:** 

Authors' roles & responsibilities	
SMY	Study conception and design; analysis and/or interpretation of data; manuscript writing; final approval of the manuscript
SLY	Analysis and/or interpretation of data; final approval of the manuscript
